# Management of uterine cystic adenomyosis by laparoscopic surgery: case report

**DOI:** 10.1186/s12905-021-01341-1

**Published:** 2021-07-01

**Authors:** Cheng-Zhi Zhao, Bin Wang, Chun-yan Zhong, Shen-tao Lu, Li Lei

**Affiliations:** 1 Department of Gynecological Pelvic Floor and Oncology, Chongqing Health Center for Women and Children, Longshan Road 120, Yubei District, Chongqing, China; 2 Department of Pathology, Chongqing Health Center for Women and Children, Longshan Road 120, Yubei District, Chongqing, China; 3 Department of Ultrasonography, Chongqing Health Center for Women and Children, Longshan Road 120, Yubei District, Chongqing, China

**Keywords:** Dysmenorrhea, Adenomyosis, Adenomyotic cyst, Laparoscopic

## Abstract

**Background:**

Endometriosis of the uterine body can be manifested as diffuse solid lesions or cystic lesions. The former is common, while the latter is rare, especially for cystic adenomyosis larger than 5 cm.

**Case presentation:**

A 30-year-old woman was admitted for severe and worsening dysmenorrhea. Ultrasound examination revealed a rare well-circumscribed cystic lesion about 5.5 × 4 × 5.0 cm. CA-125 level was slightly elevated. She accepted laparoscopic surgery and the adenomyotic tissues were excised. The histopathology of the specimen demonstrated the endometrial glands in the walls of cysts and an area of extensive hemorrhage can be seen in the inner wall of cyst. The patient made a good recovery after surgery and her symptoms complete resoluted.

**Conclusions:**

This is a rare case of a cystic adenomyotic lesion that was treated by laparoscopic surgery.

## Background

Adenomyosis is the presence of endometrial glands and stroma in the context of the myometrium, with adjacent smooth muscle hyperplasia. It may be diffuse or cystic. Diffuse adenomyosis occurs more commonly [1], and cystic adenomyosis represents a rare entity, and is more commonly encountered in younger patients [2]. Large adenomyotic cysts are lined with eutopic functional endometrium-like tissue and are characterized by cyclic changes with epithelial exfoliation and hemorrhagic infarction of adjacent smooth muscle [1]. The patients with adenomyotic cysts may have important clinical manifestations of pelvic pain, severe dysmenorrhea, and may have no any gynecologic surgical treatment.

Diagnosis and treatment of these cases pose great difficulties that will be hard to overcome until well-designed studies are launched to guide management [3].

Herein, we report our experience with a case of a cystic adenomyotic lesion that was treated by laparoscopic surgery in a 30-year-old woman.

## Case presentation

**Fig. 1:**
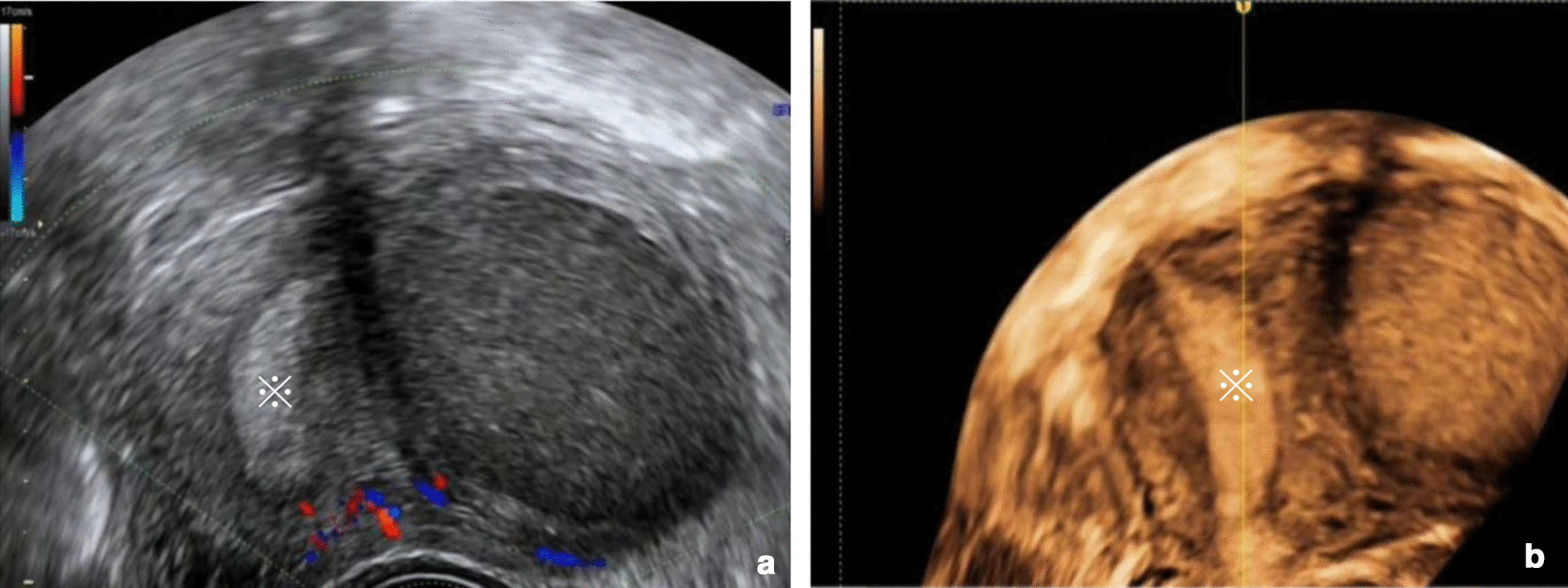
Three dimensional ultrasound images. **a** The uterus showing an normal shape of uterine cavity. **b** A well-circumscribed cystic lesion of 4.5 × 4 × 5.0 cm in the left anterior wall, and well separated from the normal uterine cavity

A 30-year-old woman was admitted for severe dysmenorrhea for approximately 2 years. She didn’t get pregnant and had no any surgical treatment. At admission, pelvic examination showed normal adnexae and an enlarged uterus. Ultrasound examination revealed well-circumscribed cystic lesion of 5.5 × 4 × 5.0 cm in the left anterior wall, separated from the normal uterine cavity (Fig. 1a, b). CA-125 level was slightly elevated (76.2 U/mL).

**Fig. 2:**
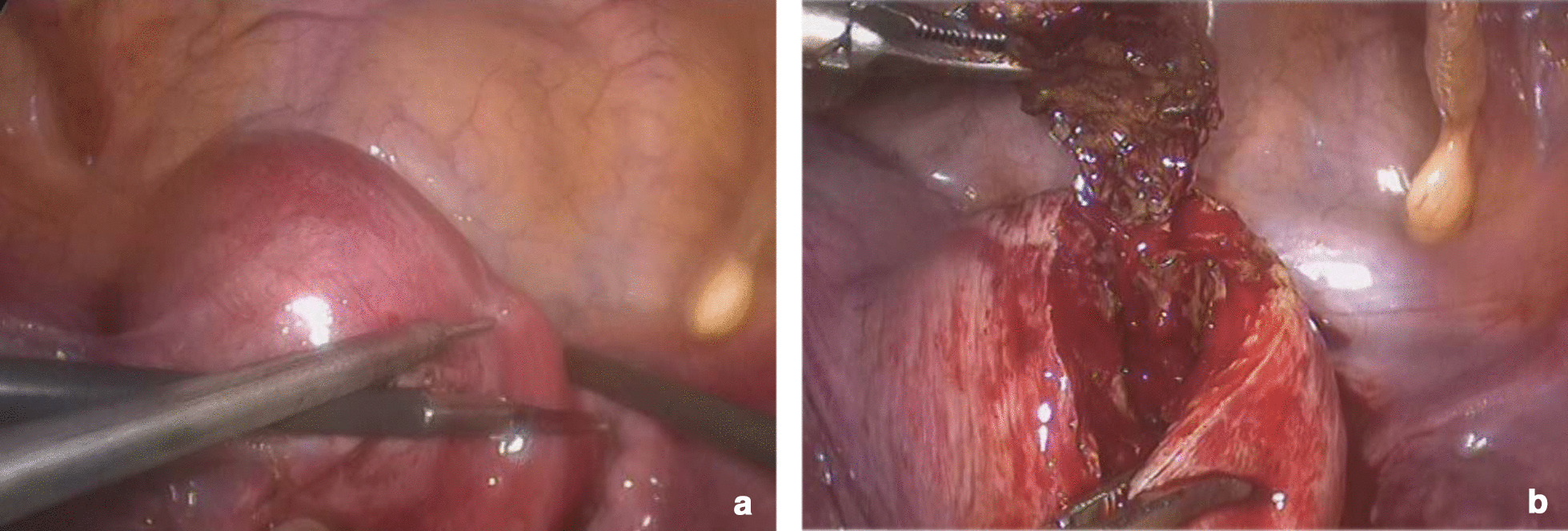
Under laparoscopic vision. **a** The uterine lesion was identified on the left portion of the uterine fundus close to the round ligament, and the uterine.** b** Chocolate-like fluid flowed from the cyst and the cystic cavity with brown tissue and no boundary with normal myoma

**Fig. 3:**
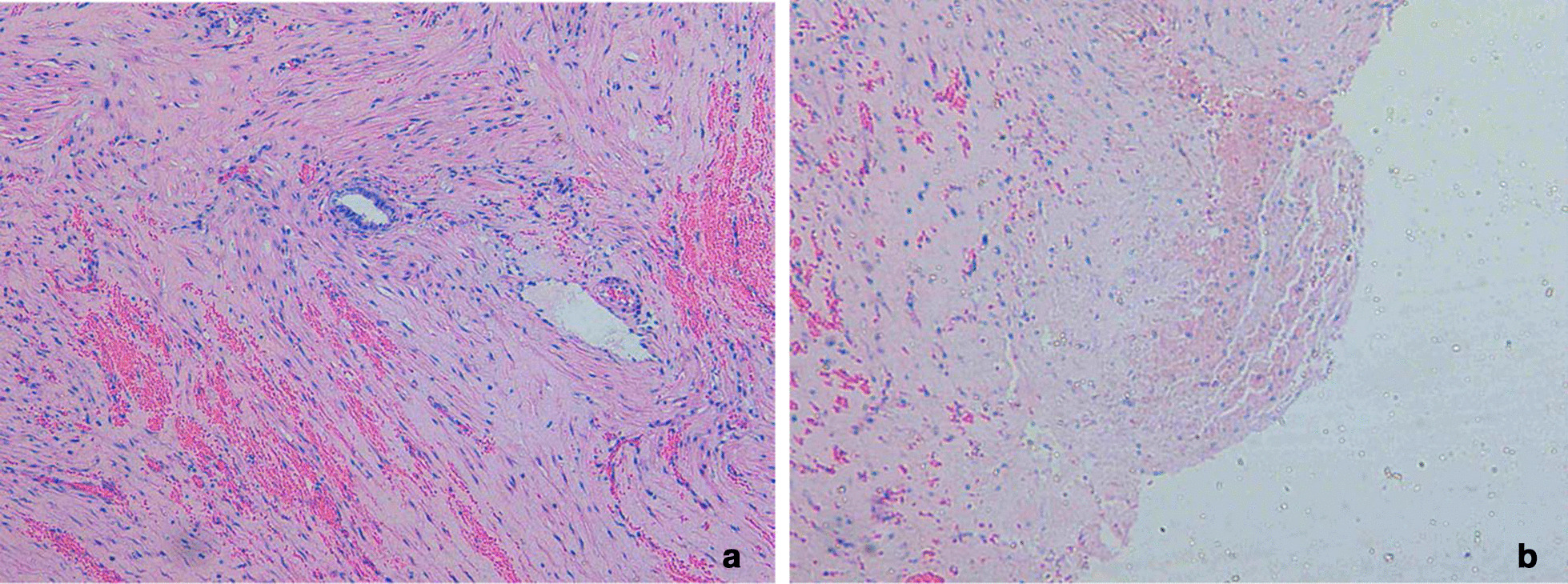
Histologic findings of adenomyotic cyst. **a** The endometrial glands lining in the walls of cysts (H&E × 40). **b** An area of extensive hemorrhage in the inner wall of cyst (H&E × 40)

**Fig. 4:**
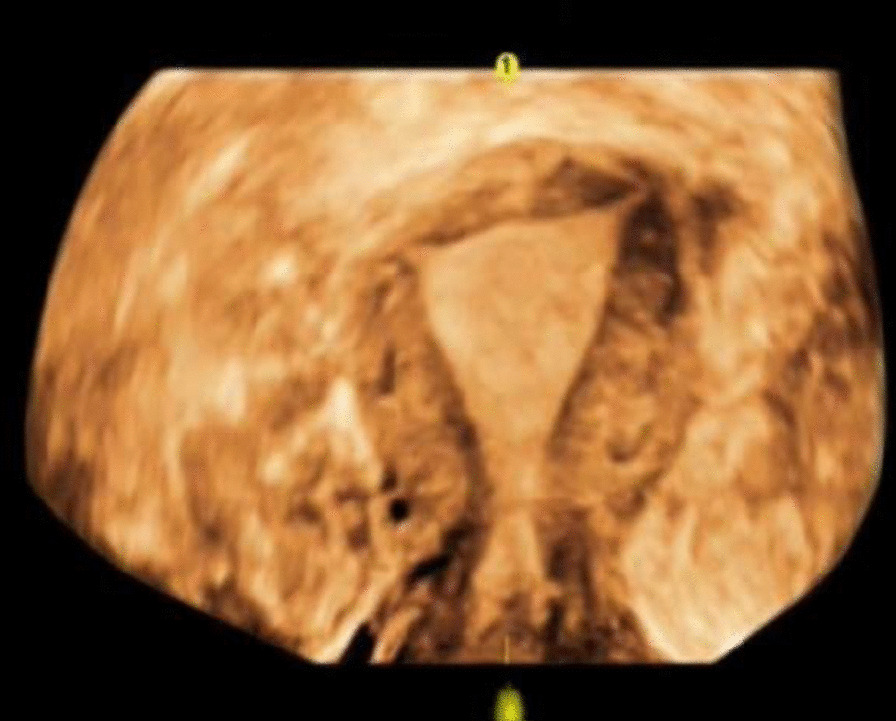
Ultrasound examination after postoperative 4 months

A minimally invasive procedure is a way of prioritizing for these diseases, so laparoscopic surgery was considered preferable for this case. At laparoscopy, the uterine lesion was identified on the left portion of the uterine fundus close to the round ligament (Fig. 2a). The ovaries and fallopian tubes appeared normal. When we opened the cystic cavity using a monopolar hook, we can see chocolate-like fluid flowed from the cyst (Fig. 2b) and a cystic cavity with brown tissue and no boundary like normal myoma (Fig. 2c). The adenomyotic tissues were excised from the surrounding myometrium, the procedure did not penetrate the uterine cavity, and the surgical wound was closed with two-layer continuous sutures (Fig. 2d). The histopathology of the specimen found the cyst wall lined by endometrial glands (Fig. 3a) and macrophages phagocytizing hemosiderin can be seen on the inner wall of cyst (Fig. 3b) confirmed the diagnosis of cystic adenomyosis [1, 2]. The patient had smooth post-operative recovery. She received the patient a single 3.75-mg dose of gonadotropin-releasing hormone (GnRH) analogue as a subcutaneous injection for three cycles, which was good to improve the effect of surgery and relieve the symptoms of dysmenorrhea after operation [1]. Her symptoms was complete resoluted on outpatient follow-up 4 months, and ultrasound examination was normal (Fig. 4) and CA125 dropped to normal (21.0 U/mL).

## Discussion and conclusions

Cystic lesion within the uterine are not common, and cystic adenomyosis are rare [4]. Uterine cysts are classified into 2 main groups: congenital and acquired. Acquired cysts include cystic degeneration of uterine leiomyoma, cystic adenomyosis, and serosal cysts. Ultrasound is the first choice for the diagnosis of adenomyosis, but MRI is more helpful for the diagnosis. Increased serum CA-125 levels have been proposed as a diagnostic tool for cystic adenomyosis. Serum CA-125 levels are generally elevated in these patients. In the present case, an extreme increase in serum CA-125 level was observed prior to surgery, which decreased after tumor removal, consistent with the previous reports.

Acién et al. [2] criteria for the diagnosis of cystic adenomyosis include (1) isolated accessory mass, (2) normal uterus (endometrial lumen), with normal Fallopian tubes and ovaries, (3) pathological examination of the surgically excised mass, (4) an accessory cavity lined by endometrial epithelium with glands and stroma, (5) a chocolate-brown-coloured fluid content, and (6) no adenomyosis (if the uterus has been removed), although there could be small foci of adenomyosis in the myometrium adjacent to the accessory cavity. In our case, the patient fulfilled all the above criteria, since histopathology of the specimen demonstrated the endometrial glands lined in the walls of cysts and macrophages phagocytizing hemosiderin can be seen on the inner wall of cyst which confirmed the diagnosis of cystic adenomyosis.

Since many patients with cystic adenomyosis are young, a minimally invasive procedure, such as laparoscopic excision, is considered preferable. Laparoscopic excision can significantly improve the associated dysmenorrhea and increase the likelihood of successful pregnancy [4]. Hormonal therapy with GnRH agonists or oral contraceptives was the therapeutic Options for cystic adenomyosis ande was somewhat effective, but the symptoms may recur again after stop of medical treatment. We given the patient a single 3.75-mg dose of gonadotropin-releasing hormone (GnRH) analogue as a subcutaneous injection for three cycles, which was good to improve the effect of surgery and relieve the symptoms of dysmenorrhea.

Cystic adenomyosis is rare. It can be asymptomatic or show progressive dysmenorrhea. Ultrasonography and MRI are complementary diagnostic tools. CA125 can be used as preoperative diagnostic index and post-operative follow up. Surgery is the preferred treatment method. GnRHa can be used as an auxiliary treatment method.

## Data Availability

All data generated or analyzed during this study are included in this published article.
